# Do You Know What I Mean? Brain Oscillations and the Understanding of Communicative Intentions

**DOI:** 10.3389/fnhum.2014.00036

**Published:** 2014-02-04

**Authors:** Marcella Brunetti, Filippo Zappasodi, Laura Marzetti, Mauro Gianni Perrucci, Simona Cirillo, Gian Luca Romani, Vittorio Pizzella, Tiziana Aureli

**Affiliations:** ^1^Institute for Advanced Biomedical Technologies, University “G. d’Annunzio” of Chieti-Pescara, Chieti, Italy; ^2^Department of Neuroscience and Imaging, University “G. d’Annunzio” of Chieti-Pescara, Chieti, Italy

**Keywords:** declarative pointing, theory of mind, social cognition, beta rhythm, gamma rhythm

## Abstract

Pointing gesture allows children to communicate their intentions before the acquisition of language. In particular, two main purposes seem to underlie the gesture: to request a desired object (imperative pointing) or to share attention on that object (declarative pointing). Since the imperative pointing has an instrumental goal and the declarative has an interpersonal one, only the latter gesture is thought to signal the infant’s awareness of the communicative partner as a mental agent. The present study examined the neural responses of adult subjects with the aim to test the hypothesis that declarative rather than imperative pointing reflects mentalizing skills. Fourteen subjects were measured in a magnetoencephalographic environment including four conditions, based on the goal of the pointing – imperative or declarative – and the role of the subject – sender or receiver of pointing. Time–frequency modulations of brain activity in each condition (declarative production and comprehension, imperative production and comprehension) were analyzed. Both low beta and high beta power were stronger during declarative than imperative condition in anterior cingulated cortex and right posterior superior temporal sulcus, respectively. Furthermore, high gamma activity was higher in right temporo-parietal junction during the sender than receiving condition. This suggests that communicative pointing modulated brain regions previously described in neuroimaging research as linked to social cognitive skills and that declarative pointing is more capable of eliciting that activation than imperative. Our results contribute to the understanding of the roles of brain rhythm dynamics in social cognition, thus supporting neural research on that topic during developmental both in typical and atypical conditions, such as autism spectrum disorder. In particular, the identification of relevant regions in a mature brain may stimulate a future work on the developmental changes of neural activation in the same regions.

## Introduction

According to cognitive pragmatics, interpersonal communication can only be successful if the partners share not just the content of an ongoing message, but also the intention. This sharing is easy to achieve when language is used, but it is more difficult when only gestures are available. If a friend of mine points at a salt bottle when looking at me, I could think that he/she wants me to pass it to him/her, or to remember the day before when I spilled the salt on the table. The gesture is the same but the intention differs: to obtain something or to share attention on something.

Pointing represents a milestone in the construction of language and meaning in early development and, indeed, it remains a crucial accompaniment of adults’ deictic speech in many languages. This gesture is typically achieved at around the end of the first year of life, when it is thought to signal the beginning of intentional communication, thereby providing the first evidence of the infant’ recognizing of the other person as endowed with mental states. In particular, two main purposes are identified as motivating the infant pointing: to request a desired object from another person (imperative pointing) or to share attention with his/her on that object (declarative pointing). The former pointing has an instrumental purpose (but, see Tomasello et al., 2007 for a broader view) and the latter an interpersonal one. A longstanding, however, lively question in developmental literature is whether only declarative pointing should be considered a true signal of early mentalizing skills (Carpenter, 2009) compared to imperative, which would merely signal the infant’s understanding of the other person as a causal instead of a mental agent.

Indeed, research data support the uniqueness of declarative pointing. Unlike the imperative, it is very rare, or even absent, in humans affected by impairment of interpersonal function, such as autism spectrum disorders (Sigman et al., 1986; Baron-Cohen, 1995; Camaioni et al., 1997); it is also absent in non-human primates, such as great apes (Call and Tomasello, 1994; Leavens et al., 2005); moreover, it is a reliable precursor of language acquisition (Desrochers et al., 1995); finally, it seems to appear a bit later in development, seeming to require more advanced socio-cognitive skills (Camaioni et al., 2004).

Very few studies on pointing can be found in neuroscience research based on the purpose of the gesture. Henderson et al. (2002), using EEG data with infants aged 14–18 months, showed that frontal regions were involved in declarative but not imperative pointing. Consistent results were also found by a PET study with the same aged infants (Caplan et al., 1993). Both studies support the hypothesis that declarative pointing is related to joint attention instead of behavior regulation.

More quantitative studies were performed on adults (Pierno et al., 2009; de Langavant et al., 2011), but neither of them showed to fully recognize the communicative nature of pointing. Pierno’s study compared BOLD signals in subjects observing an hand pointing or grasping, but found no substantial differences between the two conditions. However, the pointing the subjects were presented with had no communicative intention. In a more recent PET study, de Langavant et al. compared pointing gestures with or without a communicative function and found that right posterior superior temporal sulcus (pSTS) and right medial prefrontal cortex (mPFC) were involved in the former but not in the latter condition. However, intentions underlying communication were not distinguished. Indeed, other studies analyzing communicative abilities can be found, even if not directly referring to pointing. Human intentional communication was found to uniquely affect the brain functioning, since it showed to involve neural regions – namely the pSTS area – which were different from those involved in sensorimotor or language processes (Noordzij et al., 2009; Enrici et al., 2011). Furthermore, a magnetoencephalographic (MEG) study explored the brain dynamics involved in imperative vs. declarative communication (Vistoli et al., 2011), finding that the chronometry of neural activation at the early stage of mentalizing process showed a relatively early (before 700 ms post-stimulus) involvement of right temporo-parietal junction (rTPJ) and bilateral pSTS.

With reference to the two communicative functions emphasized by developmental literature as moving pointing gesture, i.e., declarative and imperative, the present study examined adult subjects with the MEG technique, however adapting to this environment is a task specifically devised in fMRI literature to elicit both kinds of pointing. Following the hypothesis that declarative rather than imperative pointing reflects mentalizing skills, we expected the neural network associated to those skills in adults (Saxe et al., 2004; Amodio and Frith, 2006; Becchio et al., 2006; Brüne and Brüne-Cohrs, 2006) and including pSTS, temporo-parietal junction (TPJ), precuneus and mPFC, to be active in declarative but not imperative condition. If so, the critical difference between the two kinds of pointing hypothesized by developmental research could be supported by the adult data and referred to the level of socio-cognitive engagement implied in either case.

Magnetoencephalographic recordings were used to reveal the dynamics of brain activity with high temporal resolution. Specifically, we analyzed the temporal modulation of induced oscillatory activity in the conventional physiological frequency bands (Klimesch, 1996) in a time range including both early and late latencies, the latter more likely related to high-level processes involved in social cognition. Indeed, brain rhythms are the product of synchronized activity among and within neuronal assemblies, and their power modulation is linked to sensory and cognitive functions (Wang, 2010).

To elicit communicative pointing in our sample, we employed the pointing task already used in developmental research (Camaioni et al., 2004; Aureli et al., 2009) and previously arranged for the fMRI environment (Committeri et al., 2012). This paradigm reproduces an interactive situation involving the subject and a virtual character, both of them alternatively producing or observing, pointing either for requesting an object or for sharing attention on it.

## Experimental Procedures

### Subjects

Fourteen healthy volunteers (6 females and 8 males; mean age: 26.9 ± 3.2 years; age range 22–31 years) were enrolled in the study. Inclusion criteria consisted of right-handedness as assessed with the Edinburgh inventory (Oldfield, 1971), and normal or corrected to normal vision. The exclusion criteria were: progressive neurological and/or systemic disorders; significant unstable concurrent medical illness, hormone replacement therapy; concomitant pharmacological treatment that could alter mood or cerebral metabolism (e.g., benzodiazepines, antidepressants, mood stabilizers, stimulants, or steroids) within the 30-days prior to acquisitions; a history of substance/alcohol abuse or dependence within the past 6 months (nicotine dependence was allowed); incapacities which would have limited understanding or consenting to study procedures.

All volunteers gave written informed consent according to the Declaration of Helsinki (World Medical Association Declaration of Helsinki, 1997). The protocol was approved by the local Ethic Committee (School of Medicine Ethic Committee, University of Chieti, Italy).

### Research design

The experimental paradigm was devised to reproduce a communicative setting between the subject and a virtual character. Trials were planned to allow pointing production or comprehension, with the gesture fulfilling either declarative or imperative goal. Accordingly, MEG activity was analyzed within a time window including pointing production or pointing comprehension. To improve the subject’s feeling of interacting with a real person, feedback pictures representing character’s reactions to the trials were also provided and, to prevent the subject from being bored during the session, such a feedback was either positive or negative. Since feedback pictures were introduced only for the purpose of making the interactive situation as plausible as possible, no data analysis was performed following feedback presentation.

Overall, our design comprised two sessions with declarative or imperative pointing goal. Each session included two conditions in which the subject played a producing or comprehending role.

### Materials

Home-made pictures were prepared, showing different types of images according to the four different types of communication between the subject and the character (from the subject perspective): imperative production (IP), declarative production (DP), imperative comprehension (IC), and declarative comprehension (DC).

#### Stimuli pictures

##### Production (imperative or declarative)

**Figure 1:**
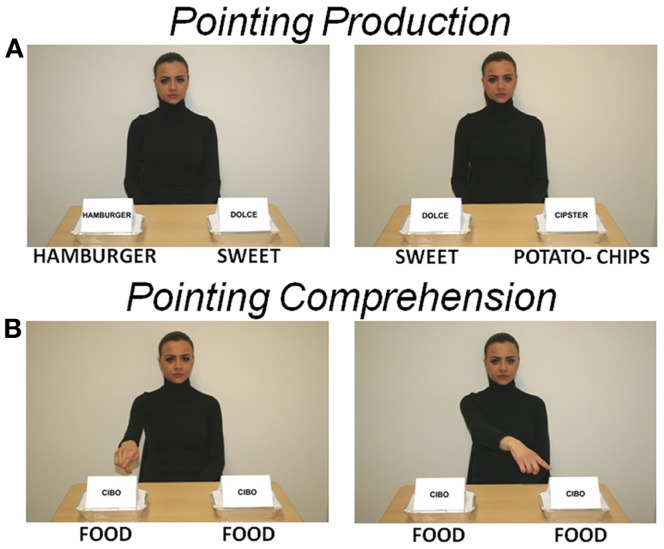
**Stimulation pictures**. The character looks at the subject. **(A)** Two take-away trays are located on the table. Each tray is marked with a label, one showing the name of a salty food (English translation has been added immediately below each picture) and the other the generic word “dolce” (sweet). Salty and sweet foods represent target and control stimuli, respectively. These pictures were used to elicit an imperative or declarative pointing on subject (POINTING PRODUCTION). **(B)** Both trays show the word “cibo” (food) and the character points with the right index finger toward either the tray on the right or on the left. These pictures were used to elicit an imperative or declarative pointing observation and comprehension on subject (POINTING COMPREHENSION). Subject passively looked at picture, no pointing had to be produced by the subject.

Twenty-four pictures were presented, showing a character – the same person who performed subject training – looking at the subject and sitting at a table on which two take-away trays were located. Both trays were marked with a label, one showing the name of a specific salty food (e.g., “hamburger,” “potato-chips”) and the other showing a generic word, i.e., “dolce” (sweet). Subjects were instructed to point only to the salty food trays, after which the feedback picture followed. The salty food was used as a target stimulus and the sweet food as a control stimulus. Salty food was located on either the left or right side in a counterbalanced random order (Figure 1A).

##### Comprehension (imperative or declarative)

Twenty-four pictures were presented. They were identical to the production pictures except for the labels on the two trays, both of them showing the generic word “cibo” (food), and for the character’s posture, showing the right index finger pointing toward either the right or left tray (Figure 1B). Subjects were instructed to observe the character’s pointing, after which the feedback picture followed.

#### Feedback pictures

##### Production (imperative or declarative)

Twenty-four (12 negative and 12 positive feedback) pictures were presented. To give the subject a feedback after producing an imperative pointing, the picture showed two hands holding either a tray containing the salty food just pointed by the subject or an empty tray, meaning positive or negative feedback, respectively. To give the subject a feedback after a DP, the figure displayed the character, sitting at a table with a tray containing the salty food just pointed by the subject, and showing a smiling or a disgusted expression, meaning that it did or did not appreciate the pointed food, respectively.

##### Comprehension (imperative or declarative)

Twenty-four (12 negative and 12 positive feedback) pictures were used. Feedback pictures to IC depicted either a tray containing the salty food pointed by the character in the previous picture, or an empty tray, meaning positive or negative feedback, respectively. Feedback pictures to DC depicted a tray containing the salty food pointed by the character in the previous picture and the character showing a neutral face expression.

All pictures were presented with Gaglab (Galati et al., 2008), an in-house software implemented in Matlab (The MathWorks, Inc., Natick, MA, USA) using Cogent Graphics toolbox (developed by John Romaya at the LON, Wellcome Department of Imaging Neuroscience, UCL, London, UK), and allowing time-locked presentation of visual and auditory stimuli with millisecond timing accuracy.

Pictures were projected by means of an LCD projector positioned outside of the shielded room. Two response boxes (Lumina, Cedrus Corporation), one for each hand, were used. Subjects’ pointing response was provided by means of the right box. At the beginning of the trial, the subject’s right index was continuously pressing a key of the right box. Pointing timing was monitored by the release of the key at the onset of the pointing movement. Pointing direction was controlled by the position (right or left) of the object that the subject had to point trial by trial. Two buttons on left box were used to interact with character during feedback.

### Experimental design

All subjects performed a training phase before the MEG session. Training was given by a female operator (the same person appearing in the stimuli/feedback pictures) and included a simulation of the experiment, which lasted until the subject felt confident in her/his ability to perform the task. Soon after, MEG measure was performed. Two main sessions, declarative and imperative, each including two conditions, production and comprehension, were used. The communicative goal of each session was conveyed to the subject at the beginning of the session and maintained throughout the session.

#### Declarative session

**Figure 2:**
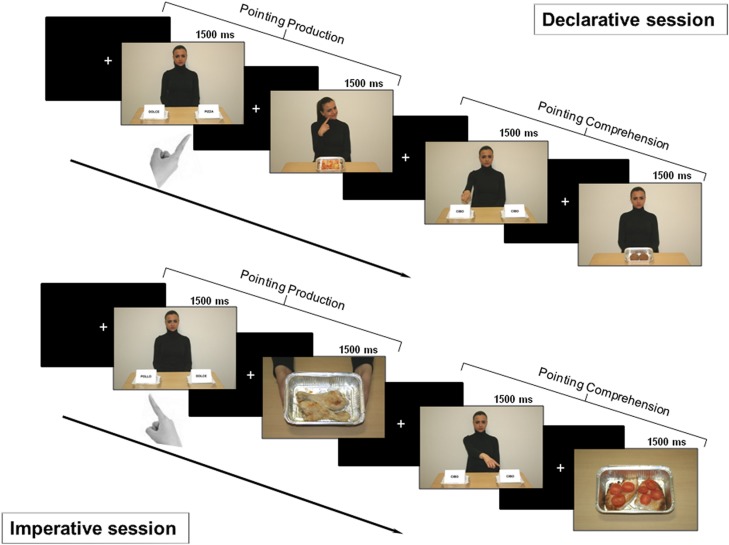
**Experimental design**. *Top: declarative session*. First part: after a fixation cross, a picture for pointing production was shown for 1500 ms. Subjects were asked to declaratively point with their right hand toward the tray containing salty food. After a variable time interval (1500/2500/3500 ms), either positive or negative feedback was provided (the character showed a smiling or disgusted expression). Second part: after a fixation cross, a picture for pointing comprehension was shown for 1500 ms. Subjects had to carefully observe the side with the food the character was pointing to, in order to provide feedback. After a time interval of variable duration, a picture displaying the food previously pointed to by the character was delivered. If the subject liked that food, she/he had to press the button at the left or the right of the panel, based on the side of the pointed food; if subject did not, no button had to be pressed. *Bottom: imperative session*. First part: after a fixation cross, a picture for pointing production was shown for 1500 ms. Subjects were asked to make an imperative pointing toward the tray containing the salty food. Then, a positive or negative feedback was given to the subject (two hands offering a tray with the pointed food or empty, respectively). Second part: after a fixation cross, a picture for pointing comprehension was shown and subjects were asked to carefully observe which tray the character was pointing to. If the food was available (full tray), the subject had to press the button corresponding to food side (left or right); otherwise (empty tray) no button had to be pressed.

In this session, we investigated pointing as a gesture for sharing intention with another person (Figure 2 – top). Therefore, the subjects were told that the task was devised either to let the character be informed about their own taste about food (production condition) or to let them informed about the character’s taste (comprehension condition).

##### Pointing production

After the presentation of a fixation cross, a picture for pointing production was shown to the subject for 1500 ms. According to previous instructions, subjects had to point to the tray marked with the label showing the name of a salty food. After a time interval of variable duration (1500/2500/3500 ms), either positive or negative feedback was given to the subject with the character showing a smiling or disgusted expression, respectively.

##### Pointing comprehension

After the presentation of a fixation cross, a picture for pointing comprehension was shown to the subject for 1500 ms. According to previous instructions, subjects had to carefully observe what tray the character was pointing to, i.e., whether it was at the right or left side. After a time interval of variable duration (1500/2500/3500 ms), a feedback picture displaying the character showing a neutral face and the food which the character pointed to in the previous picture was delivered. To have the interaction fulfilled, subjects were asked to press the left or right button of the left box, based on the side of the food previously pointed by the character, if they liked that food. Otherwise, if they did not like the pointed food, no button had to be pressed.

#### Imperative session

The session investigated pointing as a gesture for requesting something from another person (Figure 2 – bottom). Therefore, the subjects were told that the task was devised either to obtain the food by the character (production condition) or to give the food to the character (comprehension condition).

##### Pointing production

After the presentation of a fixation cross, a picture for pointing production was shown to the subject for 1500 ms. According to previous instructions, subjects had to point to the tray marked with the label showing the name of a salty food. After a time interval of variable duration (1500/2500/3500 ms), a positive or negative feedback was given to the subject by showing a picture with two hands holding either a tray with the pointed food or an empty tray, respectively.

##### Pointing comprehension

After the presentation of a fixation cross, a picture for pointing comprehension was shown to the subject for 1500 ms. According to previous instructions, subjects had to carefully observe what tray the character was pointing to, i.e., whether it was at the right or left side. After a time interval of variable duration (1500/2500/3500 ms), a picture displaying either a tray with the food pointed by the character in the previous picture or an empty tray was delivered. To have the interaction fulfilled, the subject had to press the button corresponding to the side (left or right) of the tray just pointed by the character, if the food was available (full tray). Otherwise (empty tray) no button had to be pressed.

To summarize, our paradigm included two sessions, imperative and declarative, each of them comprising 24 trials, i.e., 12 “pointing production” trials (one half displaying the target on the right of the display, and the other half on the left) and 12 “pointing comprehension” trials (one half displayed the character pointing to the tray on the right of the display and the other half on the left). Trials were randomly presented at a variable interval (a central white fixation cross on a black screen) of 1500/2500/3500 ms and were balanced with respect to condition order, target location, direction of pointing hand, and positive/negative feedback. Two runs were recorded for the declarative session and other two runs for the imperative session. In summary, four runs, each lasting about 8 min, were recorded for each MEG session, yielding 24 trials for each of the four experimental conditions (total 96 trials) for each subject. Furthermore, a set of 24 pictures was used to provide the character’s feedback (12 positive and 12 negative) in declarative as well as in imperative session.

### MEG recordings

Magnetoencephalographic signals were recorded with the 165-channel MEG system installed at the University of Chieti (Pizzella et al., 2001; Chella et al., 2012). This system includes 153 dcSQUID integrated magnetometers arranged on a helmet covering the whole head plus 12 reference channels. Two simultaneous electrical channels [electrocardiogram (ECG) and electro-oculogram (EOG)] were recorded for artifact rejection. All signals were band-pass filtered at 0.16–250 Hz and digitized at 1025 Hz.

An high-resolution MRI structural volume was acquired with a 3-T Philips Achieva MRI scanner (Philips Medical Systems, Best, The Netherlands) via a 3D fast field echo T1-weighted sequence (MP-RAGE; voxel size 1 mm isotropic, TR = 8.1 ms, echo time TE = 3.7 ms; flip angle 8°, and SENSE factor 2).

In order to coregister the head to the MRI volume, four anatomical landmarks (left and right preauricular points and nasion) were identified. Moreover, five coils were placed on the subject’s scalp and their position was recovered before and after each MEG run in order to define the subject’s head position with respect to the MEG helmet. The positions of the five coils and of the four anatomical landmarks were digitized by means of a 3D digitizer (3Space Fastrak; Polhemus).

### MEG data analysis

#### MEG source-space signal estimation

Magnetoencephalographic data were down-sampled to 341 Hz and analyzed by using an independent components analysis (ICA) approach detailed elsewhere (Mantini et al., 2011). Briefly, the algorithm automatically classifies the ICs and identifies artifactual components and components of brain origin.

The number of artifactual ICs depends on the quality of each recording. On average, the algorithm identified 12 ± 4 artifact related components. Artifact components typically included hardware or environmental-injected noise, bad channels, contamination from high noise levels, and physiologic artifacts such as magnetocardiogram, eye blinks, and movements. To determine which ICs represented artifact, a classification procedure based on: (i) IC spectral properties; (ii) IC statistical properties; and (iii) comparison of the IC time courses with the corresponding time courses of the ECG, EOG, is adopted. See Supplemental Information in de Pasquale et al. (2010) for details. A particularly important advantage of ICA based artifact rejection is that all of the 24 recorded trials for each condition and each subject are preserved, thus a reliable number of trials is maintained for the next step of the analysis. Usually, ICA based pipelines rely on the subtraction of artifactual ICs to increase the signal-to-noise ratio. Here, an alternative approach also used in other works by our group (de Pasquale et al., 2010; Betti et al., 2013; Marzetti et al., 2013) is pursued. The approach is based on reconstructing MEG signals by recombining only the ICs of brain origin. On average, the algorithm identified 15 ± 5 ICs of brain origin, each contributed by the activity of one or more dipole sources or patches (e.g., two sources with no time lag typically contribute to the same IC). Of course, in ICA based approaches a trade-off between including unwanted and excluding wanted signals has to be faced and selecting approximately 15 brain ICs is a reasonable compromise. This strategy has been shown to improve SNR in Mantini et al. (2011).

After the decomposition through the fastICA algorithm with deflation approach and the classification steps, non-artifactual IC topographies were input to the weighted minimum-norm least squares (WMNLSs) linear inverse algorithm (Fuchs et al., 1999) implemented in Curry 6.0 (Neuroscan) and the corresponding source topography was localized. In this step, the volume conductor model was given by an individual boundary element method (BEM) (Fuchs et al., 1998) and the source space was modeled by a Cartesian 3D grid bounded by the subject anatomy as derived from individual MRI.

Single subject source-space topographies were thus mapped onto a standard Montreal Neurological Institute (MNI) stereotaxic space by an affine transformation to allow spatial comparison across subjects. For each grid voxel the activity along each direction, and for each time sample, was obtained as a linear combination of non-artifactual IC time courses weighted by their related source-space topographies.

Source activity magnitude was finally derived from the Cartesian components at each voxel; i.e., square root of the sum of the squared components.

#### Regions of interest

Band limited MEG power in the whole brain was estimated by using a standard Fast Fourier Transform (FFT) approach after linear detrending and Hanning windowing. The frequency boundaries defining the physiological band ranges, i.e., theta (4–7 Hz), alpha (8–13 Hz), beta (13–30 Hz), and gamma (31–80 Hz) were based on the EEG/MEG literature (Klimesch, 1996; Neuper and Pfurtscheller, 2001; Engel and Fries, 2010; Uhlhaas et al., 2011), and confirmed by visual inspection of the averaged spectra. Stimulus locked maps for each frequency band were estimated for declarative and imperative conditions in a 500-ms time window (from 300 to 800 ms) after the stimulus image presentation. For each condition, stimulus locked power maps have been contrasted, at a group level, with the power in a baseline period (from −500 to 0 ms) prior to the stimulus image presentation. This contrast was implemented by using a paired *t*-test and the resulting maps were corrected for multiple comparisons by using the false discovery rate (Benjamini and Hochberg, 1995).

**Figure 3:**
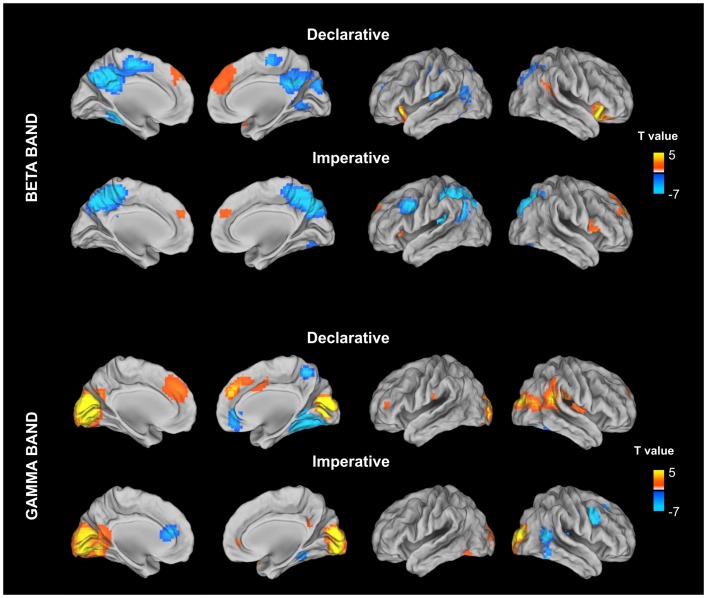
**Stimulus locked percentage relative power map in low beta band (13–20 Hz) and gamma (31–80) estimated for declarative and imperative conditions in a 500-ms time window (from 300 to 800 ms) after the stimulus image presentation**. For each condition, stimulus locked power maps have been contrasting, at a group level, with the power in a baseline period (from −500 to 0 ms) prior to the stimulus image presentation. This contrast was implemented by using a paired *t*-test and the resulting maps were corrected for multiple comparisons by using the false discovery rate (FDR).

This procedure led to the identification of 17 ROIs showing a significant modulation with respect to the baseline in either the declarative or imperative condition in beta and gamma bands (see Table 1; Figure 3).

**Table 1:** **Montreal Neurological Institute coordinates of the maximum activity peak for regions of interest showing significant modulations of band power with respect to baseline (FDR corrected)**.

Region of interest	MNI coordinates			Frequency band
	*x*	*y*	*z*	
ACC	6	40	28	β, γ
lOFC	−33	22	−3	β
rOFC	34	19	−10	β
rSFg	15	50	44	β
lPMc	−43	12	48	β
rMFg	40	9	43	γ
rSMA	4	−21	62	β
lpIPS	−25	−62	51	β
rpIPS	25	−64	51	β
lIPS	−33	−54	47	β
rPCC	7	−47	33	β
lAg	−47	−62	17	β
rTPJ	60	−49	28	γ
rpSTS	58	−443	15	β
lpIC	−34	−29	15	β
rCn	4	−80	28	β, γ
rPCn	6	−54	60	β, γ

*ACC, anterior cingulate cortex; lOFc/rOFc, left/right orbitofrontal cortex; rSFg, right superior frontal gyrus; lPMc, left premotor cortex; rMFg, right middle frontal gyrus; rSMa, right supplementary motor area; lpIPS/rpIPS, left/right posterior intraparietal sulcus; lIPS, left intraparietal sulcus; rPCC, right posterior cingulate cortex; lAg, left angular gyrus; rTPJ, right temporo-parietal junction; rpSTS, right posterior superior temporal sulcus; lpIC, left posterior insular cortex; rCn, right cuneus; rPCn, right precuneus*.

### Time–frequency analysis

Time–frequency analysis allowed us to investigate whether the four different experimental conditions induced changes of band power in the identified ROIs. To this end, time–frequency representations (TFRs) were estimated in a 1500-ms time window starting 250 ms prior to the image presentation. Specifically, TFRs for each ROI were calculated by Morlet wavelets (Tallon-Baudry et al., 1997; Jensen et al., 2002). The power at a given time *t* and frequency *f*_0_ is thus provided by the squared value of the convolution of the signal to a Morlet’s wavelet:

(1) $$\psi \left(t,\;f_{0}\right)=Ae^{-\frac{t^{2}}{2\sigma_{t}^{2}}e^{2\pi if_{0}t}}$$

where *A* = (σ*_t_*√π)^−1/2^. The wavelet width was set to *f*_0_2πσ*_t_* = 7 as a balance between temporal and frequency resolution (Jensen et al., 2002). Accordingly, power modulation was evaluated by calculating relative changes in TFR of power after the stimulus image presentation (Pow) with respect to mean power in a baseline period of 200 ms (from −250 to −50 ms) prior to the stimulus image presentation (Pow_bas_) for each experimental condition separately. To this aim, event-related synchronization (ERS) or event-related desynchronization (ERD) (Pfurtscheller and Lopes da Silva, 1999; Neuper and Pfurtscheller, 2001) values were obtained according to:

(2) $$\mathrm{ERD}∕\mathrm{ERS}\;=100\frac{\left(\mathrm{Pow}-\mathrm{Po}w_{\mathrm{bas}}\right)}{\mathrm{Po}w_{\mathrm{bas}}}$$

Since a high variability in reaction times (RTs) of pointing response across subjects was observed (RTs ranging from 737 to 1332 ms), a normalization of time epochs was performed in order to realign the timing of brain processes underlying the pointing production task. To this aim, for each subject the TFR plot between 100 ms after the stimulus and the individual RT was referenced to a standard time interval between 100 and 900 ms (mean value of the RTs across subjects in the different conditions), by means of a cubic spline interpolation. Consequently, epochs shorter than 900 ms were stretched and those longer than 900 ms were shrunk to fit the standard time epoch. Henceforth, in the TFR plots the motor response occurs exactly at 900 ms. Furthermore, concerning the pointing comprehension task (in which RTs were absent since no pointing response but only pointing observation was required), an analog time window analysis (between 100 and 900 ms after “pointing comprehension stimulus” onset) was applied to analyze the pointing observation processing.

To test the effect of the different conditions on rhythmic activity modulation over time, the whole normalized time epoch was partitioned into five-time intervals. Mean relative power values were estimated by averaging TFR values in a given time interval (*t*_1_ = 101–260 ms; *t*_2_ = 261–420 ms; *t*_3_ = 421–580 ms; *t*_4_ = 581–740 ms; *t*_5_ = 741–900 ms) and physiological frequency band for each ROI and condition. The frequency boundaries defining the physiological bands were chosen according to the EEG/MEG literature (Klimesch, 1996; Neuper and Pfurtscheller, 2001; Engel and Fries, 2010; Uhlhaas et al., 2011): theta (4–7 Hz), alpha (8–13 Hz), low beta (13–20 Hz), high beta (21–30 Hz), low gamma (31–50 Hz), and high gamma (51–80 Hz).

### Statistical analysis

#### Behavioral data

Reaction time of pointing motor response was analyzed by a one-way ANOVA to identify possible statistical differences within the two communicative goals (declarative vs. imperative). Statistical analysis was performed using Statistica 6.1 software (Statsoft Italia Srl 2003).

#### MEG data

To assess specificity of ROI reactivity differences between declarative and imperative conditions, we performed a repeated measures ANOVA with *Goal* (declarative, imperative) × *Band* (beta, gamma) × *ROI* (listed in Table 1). A triple interaction was found [*F*(16,208) = 1.948, *p* < 0.02], indicating specific regional differences between conditions. To further test the effect of conditions on rhythmic activity modulation over time, a repeated measures ANOVA was performed separately for each frequency band and ROI on the time interval averaged TFR values, with *Role* (production, comprehension), *Goal* (declarative, imperative), and *Time* (*t*_1_, *t*_2_, *t*_3_, *t*_4_, *t*_5_) as within subject factors. Bonferroni *post hoc* test was applied when significant interactions were found. Statistical analysis was performed using Statistica 6.1 software (Statsoft Italia Srl 2003).

## Results

### Behavioral data

No significant differences were found for RTs between goals (one-way ANOVA with *Goal* – declarative and imperative – as within subject factor); mean ± SD: declarative RT: 889 ± 113 ms, imperative RT: 924 ± 163 ms.

### MEG data

The assessment of the differences between rhythmic activity modulations across conditions in the ROIs by means of ANOVA showed significant effects only for the medial anterior cingulate cortex (ACC), the rTPJ, and the right posterior superior temporal sulcus (rpSTS). These effects were observed in specific frequency bands.

**Figure 4:**
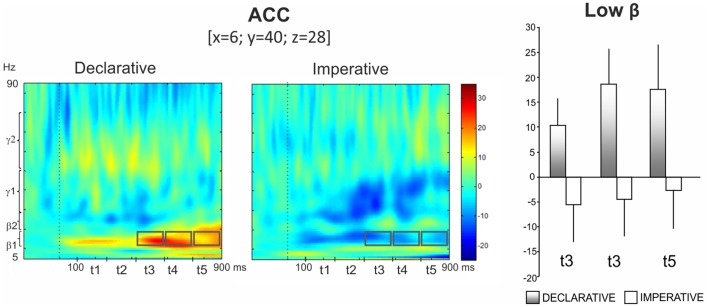
**Anterior cingulate cortex (ACC)**. Group TFRs during declarative and imperative sessions: dotted line indicates the stimulus onset (left). Gray boxes indicate the frequency bands and time intervals where a significant effect of Goal was found in Bonferroni *post hoc* test. Bar plots show average TFR values within gray boxes together with their standard deviations (right). Significant differences as revealed by Bonferroni *post hoc* test were found in *t*_3_ (*p* < 0.01), *t*_4_ (*p* < 0.001) and *t*_5_ (*p* < 0.001).

In particular, the repeated measures ANOVA revealed a significant *Goal* effect in low beta for ACC, with the activity being higher in the declarative rather than the imperative session [*F*(1,13) = 8.10, *p* < 0.02]. Furthermore, a significant *Goal* × *Time* interaction was observed [*F*(4,52) = 3.81, *p* < 0.01], indicating a time specific difference of power modulation between the declarative and the imperative sessions. Bonferroni *post hoc* test revealed that this difference was significant at the *t*_3_ (*p* < 0.01), *t*_4_ (*p* < 0.001) and *t*_5_ (*p* < 0.001) (421–580, 580–740, 740–900 ms, respectively; Figure 4, right panel) time intervals. Indeed, Figure 4 (left panel) shows a sustained low beta power modulation after the stimulus onset lasting until the motor response. Low beta power increased during the declarative and decreased during the imperative condition, regardless of the subject role. Furthermore, the difference between declarative and imperative low beta power increased with time and was maximal in the last time interval.

**Figure 5:**
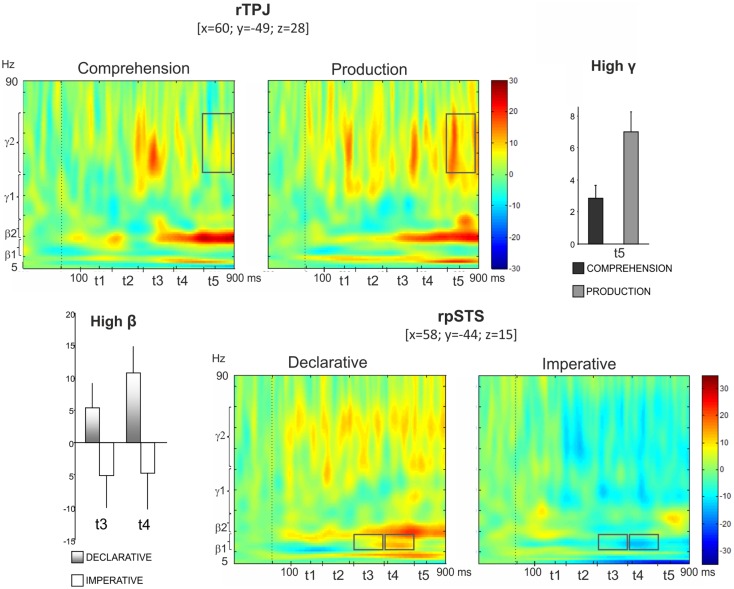
***Top*: right temporo-parietal junction (rTPJ)**. Group TFRs during pointing comprehension and production: dotted line indicates the stimulus onset (left). In the frequency and time interval marked by superimposed gray boxes (740–900 ms, high gamma), a time specific difference of power modulation between the production and the comprehension sessions was found. Bar plots show results for Bonferroni *post hoc* test: *p* < 0.01 (right). *Bottom*: right posterior superior temporal sulcus (rpSTS). Group TFRs during declarative and imperative sessions (right). Significant increased high beta power during the declarative condition as well as a decreased high beta power during the imperative condition were observed in the frequency and time intervals marked by superimposed gray boxes. Bonferroni *post hoc* evidenced a significant difference during *t*_3_ (*p* < 0.05) and *t*_4_ (*p* < 0.01) (left).

Figure 5 (top left panel) shows power modulations for rTPJ during both comprehension and production conditions. Repeated measures ANOVA revealed a *Role* main effect [*F*(1,13) = 9.87, *p* < 0.05] in high gamma, the power increase being higher during pointing production than comprehension. Furthermore, a *Role* × *Time* interaction was observed [*F*(4,52) = 3.52, *p* < 0.05], indicating a time-specific difference of power modulation between the production and the comprehension sessions. In fact, Bonferroni *post hoc* revealed a significant difference during *t*_5_ (*p* < 0.01) (Figure 5, top right panel).

Furthermore, the bottom right panel of Figure 5 shows TFR plots for rpSTS during both declarative and imperative conditions. High beta power showed an increase during the declarative condition and a decrease during the imperative condition. This effect was specific for the *t*_3_ and *t*_4_ time intervals. Indeed, the repeated measures ANOVA revealed a *Goal* × *Time* significant interaction in high beta [*F*(4,52) = 2.96, *p* < 0.05] and the Bonferroni *post hoc* resulted in a significant difference during *t*_3_ (*p* < 0.05) and *t*_4_ (*p* < 0.01); (Figure 5, bottom left panel).

In addition to these significant results, a tendency to a significant *Goal* effect (*p* = 0.084) was found in the low gamma band (31–50 Hz) for ACC.

## Discussion

This study used the MEG technique to analyze brain rhythms underlying comprehension and production of pointing as a function of communicative intention, either imperative or declarative. We adapted an interactive task previously used in developmental research (Camaioni et al., 2004; Aureli et al., 2009) and arranged for the fMRI environment (Committeri et al., 2012), with the subject playing alternatively the role of the sender or the addressee of the gesture in two different communicative contexts. The aim was to bring in the subject in a more ecological situation than that usually provided by neuroimaging studies and more similar to that used in social cognition research (Hari and Kujala, 2009). Furthermore, for the first time as far as we are aware, we analyzed the induced oscillatory activity on data elicited by a social cognition task. This strategy allows for the observation of rhythmic modulations of the activity of specific ROIs in a time range which includes late latencies, possibly related to the high-level processes involved in social cognition (Wang et al., 2010). Our results showed different frequency-specific modulations of power based on both the goal of the gesture and the role of the subject.

### Declarative vs. imperative goal of pointing

With respect to the goal of the gesture, in low beta, the declarative pointing elicited higher activity than imperative pointing in dorsal ACC, starting from 421 to 900 ms after the stimulus onset (i.e., the last three periods before motor response). This region is included in the medial frontal cortex (MFC), which was consistently found to play a core role in social cognition. In particular, Amodio and Frith (2006) argued for a functional subdivision of rostral MFC in an anterior and a more posterior section: the former associated with self-knowledge and emotional processing (Lieberman et al., 2004; Steele and Lawrie, 2004; Stern et al., 2009), attribution of mental states (Mitchell et al., 2005), and mentalizing (Brunet et al., 2000; Gallagher et al., 2000; Frith and Frith, 2003; Uchiyama et al., 2012); the latter associated to cognitive processing such as internal action monitoring and decision making (Botvinick et al., 2004; Walton et al., 2004). In this framework, the region identified by our study was localized along this anterior/posterior border of ACC and was activated in the declarative condition, regardless of the subject role (comprehension or production).

A greater beta band functional connectivity between the frontal and right temporal areas during unfamiliar compared to familiar display was showed (Calmels et al., 2012). Furthermore, beta band activity in this cortical area was also recognized as being involved in several cognitive activities, such as learning after positive feedback (van de Vijver et al., 2011), decision making (Cohen et al., 2007), and reward processing (Marco-Pallares et al., 2008). More specifically, the last two studies observed by means of EEG, a reward-related oscillatory activity between 20 and 30 Hz, highlighting the advantage of the use of time–frequency analysis to observe high cognitive processing dynamics. Results from these studies demonstrate that reward processing in the preceding trials could affect oscillatory activity on the next trials. These results were obtained by adequately manipulating positive and negative feedback preceding each trial, and by assigning them a “winner/looser” value. On the contrary, feedback used in our study was devoid of a right/wrong value, since it had the purpose to support the joint attention, over than to prevent the subject from being bored during the session, rather than functioning as a penalty/reward. Consequently, an interpretation of our data in terms of rewards could be speculative.

All these findings suggest a top-down role of beta oscillatory activity in MFC. In particular, Engel and Fries (2010) hypothesize that beta band activity is related to the maintenance of a given cognitive status and that the enhancement of that activity reflects the endogenous vs. exogenous components of subject performance. Looking at our results, we suggest that the enhanced late latency beta band activity, found in ACC-arMFC when the subjects were presented with declarative condition, reflects the endogenous components required by the mentalizing attitude involved in that condition. Conversely, the decreased beta band power, presumed to be involved in causal processes, could reflect exogenous components that interrupted the current cognitive setting during imperative condition.

A second finding concerned the right posterior STS activation in the 20- to 30-Hz frequency range during declarative pointing, in the time interval from 421 to 740 ms post-stimulus. Previous fMRI study demonstrated the role of pSTS in passive viewing of biological motion (Pelphrey et al., 2003), in the extraction of social cues like directional eye gaze (Materna et al., 2008a,b), and in intentional action understanding (Pelphrey et al., 2004). Furthermore, a MEG study by Vistoli et al. (2011) observed a right posterior STS activity 200–600 ms after the stimulus presentation during an intention attribution task. Finally, de Langavant et al. (2011) showed, by means of PET data, the right posterior STS involvement in communicative vs. non-communicative pointing. Our data confirmed the involvement of this region in social interaction when attention is shared with another person as it happens in the declarative condition. Moreover, the observed beta ERS at later latency extends to this region, the above hypothesis on beta band, as reflecting the activity of an endogenous, top-down mechanism.

In conclusion, both right posterior STS and MFC seemed to work in social cognition as high-level processing sites, since they seem to be selectively involved in intentions underlying the declarative pointing. This function was carried out according to different temporal dynamics, since the prefrontal area maintained a sustained activity, whereas right posterior temporal area showed a time-limited modulation. This suggested that a partial overlap in time, at a middle stage, between these two regions underpins social cognition.

### Sender vs. receiver role in pointing

The second goal of our study was to analyze the brain oscillatory activity based on the subject role; i.e., as sender vs. receiver of the pointing gesture. The most important result was provided by the time–frequency modulation in the rTPJ, which showed significant high gamma band ERS during pointing production and not during pointing comprehension, in the 160–0 ms time interval before movement.

Several studies have suggested rTPJ as a critical core for comparing information coming from a self-produced action with those from the environment. Specifically, TPJ has been considered the region which was mostly engaged when the individuals had to distinguish between themselves and the others (Decety and Sommerville, 2003; Uddin et al., 2006), thus playing a predominant role in the sense of agency (Ruby and Decety, 2001; Farrer et al., 2003; Sperduti et al., 2011). The TPJ role was also addressed by Corbetta et al. (2008) with reference to the theory of mind (ToM) ability. According to the authors, TPJ activity could work as an important tool for switching between internal and external signals in the comparison of the self and the other. Our results, showing a gamma band TPJ ERS at a late latency during pointing production regardless of the intention of communication, could suggest a TPJ involvement in agency function. Moreover, since TPJ activation started after the accomplishment of other high-level mental activities localized in posterior STS, such as social information and communicative intention processing (see also Materna et al., 2008a), our data are in accordance with the Decety and Lamm’s (2007) quantitative meta-analysis, revealing that the TPJ activation overlaps in different cognitive domains both at high and low level. These data support the evolutionary hypothesis proposed by these authors, according to which high-level mechanisms operate on functionally more primitive levels.

As an additional result, we found that the involvement of TPJ during pointing production was expressed by an enhanced gamma band power. Previous research showed that this frequency range is associated with attention (Jensen et al., 2007), movement preparation (Schoffelen et al., 2011), and conscious awareness (Meador, 2002). Furthermore, this rhythm was observed in the monkey lateral intraparietal area (the putative homologous of human IPS/SPL) during the delay of a delayed saccade task (Pesaran et al., 2002). Finally, gamma band synchronization in parietal cortex was considered to represent the planned direction of the saccade (Van Der Werf et al., 2008). These data, together with the lack of motor responses during pointing comprehension – that prevents from excluding that TPJ activation can be ascribed to motor planning – could drive a possible confound in the TPJ role interpretation. Nevertheless, a previous PET study provided evidence for an increased rCBF in the posterior part of the right STS, close to the TPJ, during communicative pointing contrasted to uncommunicative pointing (de Langavant et al., 2011). The absence of activation in this region during a motor response devoid of communicative intention strongly supports a TPJ involvement in agency rather than in a mere motor process. Interestingly, induced gamma band bursts were observed in 4-month-old infants in right posterior areas (occipital, temporal and parietal EEG channels; Grossmann et al., 2007). This oscillation was elicited during averted vs. directed gaze observation. Averted gaze assumes a central role during social communication by directing the perceiver’s attention toward a location. The authors suggest that their finding of gamma burst in response to averted gaze might reflects a shift in spatial attention, highlighting the potential role played by gamma band oscillations in examining the development of social perception.

Taken together, these studies on adults and infants seem to support the interpretation of enhanced rTPJ gamma oscillations observed in our study during pointing production as a sign of the involvement of this region in switching between internal and external aspects and between different spatial locations. In other words, pointing production would require the individual to decide between objects sited in two spatial locations and at the same time to plan a communicative action (pointing) in order to direct the other’s attention toward the pointed location.

An alternative view comes from the recent meta-analysis by Kubit and Jack (2013). The authors hypothesize that the overlap of attention reorienting and social cognition on the same cortical region (rTPJ) could be viewed in a new light. They consider the presence of two distinct regions (angular gyrus and supramarginal gyrus) close to rTPJ that could mutually inhibit their activity in response to non-social vs. social tasks. Nevertheless, the comparison between social and non-social tasks is beyond the scope of this work. An *ad hoc* paradigm should be designed to address this issue.

In conclusion, we found that the core of the ToM circuit is active during declarative but not during imperative pointing. This finding suggests that declarative pointing reflects mentalizing skills, thus confirming in human adults the difference between imperative and declarative pointing hypothesized in developmental research. Furthermore, our results suggest that a complex process, such as a communicative interaction, involves a distributed neural circuit in which the modulation of oscillatory activity of different regions is partially overlapped in time. This process may run in parallel with a functional, effect-specific, differentiation between right temporal region and frontal areas, ending in the parietal and medial frontal regions. Our results contribute to the understanding of the roles of brain rhythm dynamics in social cognition, potentially opening the way for a targeted investigation of social interaction and language precursors during development, as well as of alteration of normal social abilities such as those related to autism spectrum disorder. The identification of relevant brain areas and, more importantly, of the frequency bands and the timing of the activation of these regions may greatly contribute to a future work on development, by suggesting to monitor the neural changes occurring in the same regions before the mature form is achieved.

## Conflict of Interest Statement

The authors declare that the research was conducted in the absence of any commercial or financial relationships that could be construed as a potential conflict of interest.
